# The blood-borne miRNA signature of lung cancer patients is independent of histology but influenced by metastases

**DOI:** 10.1186/1476-4598-13-202

**Published:** 2014-08-30

**Authors:** Petra Leidinger, Christina Backes, Michael Blatt, Andreas Keller, Hanno Huwer, Philipp Lepper, Robert Bals, Eckart Meese

**Affiliations:** Department of Human Genetics, Medical School, Saarland University, Building 60, Homburg/Saar, 66421 Germany; Department of Pneumology, Medical School, Saarland University, Building 91, Homburg/Saar, 66421 Germany; Department of Clinical Bioinformatics, Saarland University, Building E2.1, Saarbrücken, 66123 Germany; Department of Thoracic Surgery, Voelklingen Heart Center, Voelklingen, 66333 Germany

**Keywords:** MicroRNA, Microarray, Expression profile, Blood, Histology, Lung cancer, Small cell lung cancer, Non-small cell lung cancer, Adenocarcinoma, Squamous cell lung cancer, Metastasis

## Abstract

**Objectives:**

In our previous studies we reported a panel of 24 miRNAs that allowed discrimination between blood of lung tumor patients independent of the histological subtype and blood of healthy controls with an accuracy of 95.4% [94.9%-95.9%]. Here, we now separately analyzed the miRNA expression in blood of non-small cell lung cancer (NSCLC), including squamous cell lung cancer and adenocarcinoma, and small cell lung cancer (SCLC) patients.

**Patients and methods:**

In total, we examined the expression levels of 1,205 miRNAs in blood samples from 20 patients from each of the three histological groups and determined differentially expressed miRNAs between histological subtypes and metastatic and non-metastatic lung cancer. We further determined the overlap of miRNAs expressed in each subgroup with the 24-miRNA signature of lung tumor patients.

**Results:**

Based on a raw p-value < 0.05, only 18 blood-borne miRNAs were differentially expressed between patients with adenocarcinoma and with squamous cell lung carcinoma, 11 miRNAs between adenocarcinoma and SCLC, and 2 between squamous cell lung carcinoma and SCLC. Likewise, the comparison based on a fold change of 1.5 did not reveal major differences of the blood-borne miRNA expression pattern between NSCLC and SCLC. In addition, we found a large overlap between the blood-borne miRNAs detected in the three histological subgroups and the previously described 24-miRNA signature that separates lung cancer patients form controls. We identified several miRNAs that allowed differentiating between metastatic and non-metastatic tumors both in blood of patients with adenocarcinoma and in blood of patients with SCLC.

**Conclusion:**

There is a common miRNA expression pattern in blood of lung cancer patients that does not allow a reliable further subtyping into NSCLC or SCLC, or into adenocarcinoma and squamous cell lung cancer. The previously described 24-miRNA signature for lung cancer appears not primarily dependent on histological subtypes. However, metastatic adenocarcinoma and SCLC can be predicted with 75% accuracy.

**Electronic supplementary material:**

The online version of this article (doi:10.1186/1476-4598-13-202) contains supplementary material, which is available to authorized users.

## Background

Lung cancer is worldwide the leading cause of cancer related deaths in both men and women with estimated 1,608,055 (12.72%) new cases and 1,376,579 (18.2%) cancer deaths in 2008 ([1] available from: http://globocan.iarc.fr, accessed on 09/01/2013). Primarily, lung cancer is divided into two main histological subtypes depending on their cells of origin. Non-small cell lung cancer (NSCLC) account for about 80% of all lung cancers and are further divided by their origin into adenocarcinoma (Adeno-Ca, about 40%), squamous cell carcinoma (sqCLC, about 30%) and large cell carcinoma (about 9%). Small cell lung cancer (SCLC) is far more aggressive than NSCLC and accounts for about 15% of all lung cancers. The classification into the different histological lung cancer types plays a prominent role in clinical management and prognosis of the disease [2].

As most SCLC have spread to other parts of the body at the time of diagnosis surgery is often ineffective, but they respond well to chemotherapy and radiation. For early stage I and II NSCLCs, surgery is the treatment of choice. However, as these two stages combined account for only 25 to 30% of all patients with lung cancer the most common treatment is systemic therapy (chemo- or targeted therapy) and/or radiotherapy [3–5]. Among NSCLCs, adenocarcinoma and squamous cell carcinoma differ with regards to the clinical management and the prognosis. Adenocarcinoma are, for example, more likely to metastasize to the lymph nodes and the brain than squamous cell lung cancer [6]. Lung cancer diagnosis and subclassification is normally based on light microscopic criteria but an accurate diagnosis of histological lung cancer subtypes is often hampered by small tissue biopsies and high observer variability [7–9].

Since several years, microRNAs (miRNAs) have shifted more and more into focus as cancer biomarkers. MiRNAs are small non-coding RNA molecules that are involved in many physiological and pathological processes due to their ability to regulate the expression of most human genes. In the recent past, it was shown that miRNAs are tissue specific and suitable to classify human cancers [10, 11]. Especially for lung cancer it was recently shown that classification of different subtypes due to the miRNA expression pattern is possible [12–15]. While these studies were done on lung cancer tissue or cells, there is an increasing number of investigations on miRNAs in the circulation as potential biomarkers [16–27]. In our previous studies, we were able to show that blood-based miRNA expression profiles differentiate between lung cancer patients, patients with COPD and healthy individuals [24, 26]. While these studies show that various diseases including lung cancer can clearly be differentiated from controls by a blood-borne miRNA pattern, there is significant less evidence that histological subtypes of a disease can also be classified by blood-borne miRNA expression pattern. Here we set out to analyze the miRNA expression of different histological lung cancer subtypes. In detail, we investigated the miRNA expression profile of blood from patients with non-small cell lung cancer, including squamous cell lung cancer and adenocarcinoma, and small cell lung cancer.

## Results

### Overall miRNA expression in blood of lung cancer patients

**Figure 1:**
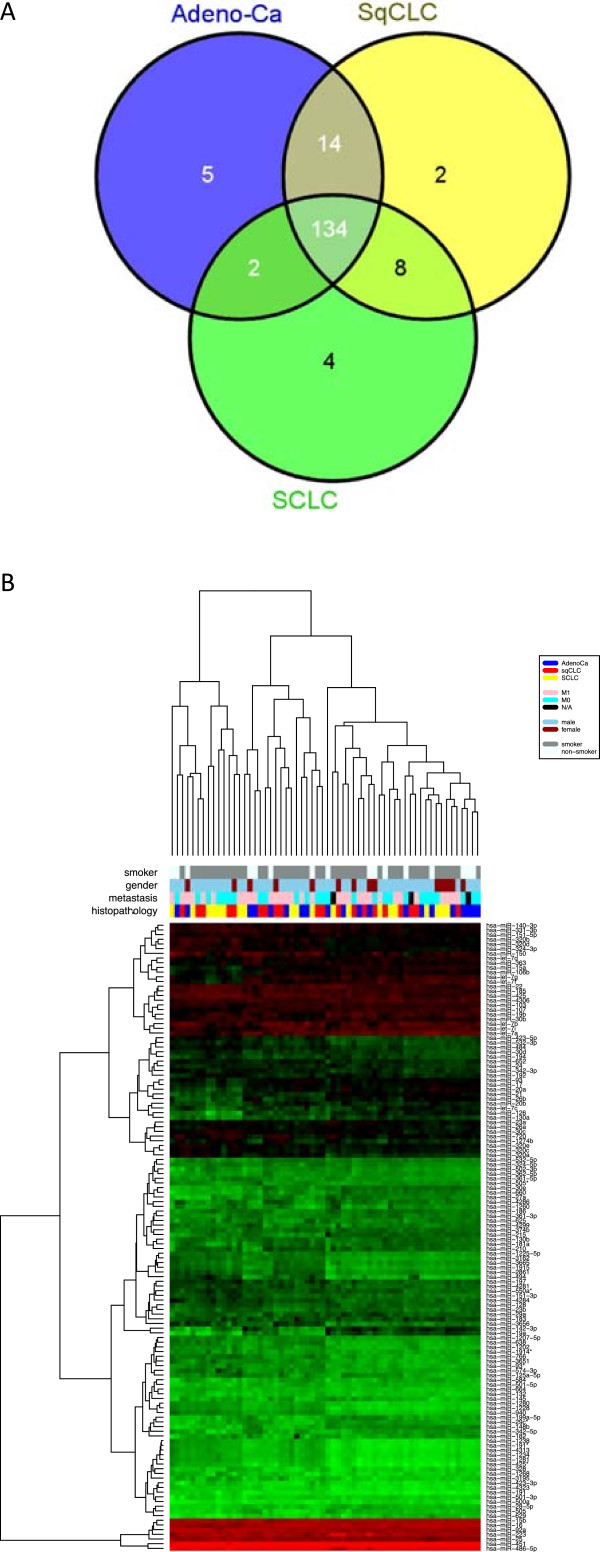
**Overall miRNA expression in blood of lung cancer patients. A**: Venn-diagram of detected miRNAs per group. Each circle of the three-way Venn-diagram indicates the numbers of miRNAs that are detected in whole blood samples of either all 20 adenocarcinoma patients, all 20 squamous cell lung cancer patients, or all 20 SCLC patients. Adeno-Ca = adenocarcinoma, sqCLC = squamous cell lung cancer, SCLC = small cell lung cancer. **B**: Heatmap of the 134 miRNAs detected in all analyzed 60 patient samples. Each column represents a blood sample and each row represents a miRNA. The miRNA expression values are visualized by the red-green color code, where green means low expression and red means high expression. The colored bars under the dendrogram indicate the histological subtype, the smoking status, the gender, and the metastatic state of the respective samples (red = squamous cell lung cancer (sqCLC), blue = adenocarcinoma, yellow = SCLC, pink = metastasis (M1a or M1b), cyan = no metastasis (M0), black = no information on metastasis state, skyblue = male, darkred = female, grey = current or former smoker, white = never-smoker).

Previously, we reported a panel of 24 blood-borne miRNAs that allowed discrimination between lung tumor patients and healthy controls [24]. Here, we analyzed the expression of blood-borne miRNAs in patients with NSCLC (squamous cell lung cancer, adenocarcinoma) or SCLC. In detail, we examined the expression levels of 1,205 miRNAs in whole blood samples collected in PAXgene blood RNA tubes. RNA expression profiles were generated from 20 patients with SCLC and 40 patients with NSCLC including 20 adenocarcinoma and 20 squamous cell lung carcinoma. Of the 1,205 analyzed miRNAs, 872 miRNAs were not detected in any adenocarcinoma sample, 868 miRNAs not in squamous cell lung cancer samples and 853 miRNAs not in SCLC samples. Besides these non-detected miRNAs, we found large numbers of miRNAs that were detected in all samples of each group. Specifically, we found 155 miRNAs that were detected in all 20 adenocarcinoma samples, 158 miRNAs in all squamous cell lung cancer samples, and 148 miRNAs in all SCLC samples. Notably, we found a high overlap of 134 miRNAs that were expressed in all 60 samples. The detected miRNAs per group and the overlaps between the groups are shown in Figure 1A. Figure 1 B shows the heatmap of the unsupervised hierarchical clustering based on the 134 miRNAs detected in all samples.

### Differential miRNA expression profiles in blood of patients with histological different lung cancer types

To further analyze the similarity/difference between the miRNA pattern of the subtypes, we performed two-tailed unpaired t-tests for each of the 1,205 miRNA and all samples of each of the three subgroups. After adjusting the p-values by the Benjamini-Hochberg approach, we did not find any significantly deregulated miRNAs (p < 0.05). Without adjustment, we found only 18 miRNAs differentially expressed between blood samples of patients with adenocarcinoma and patients with squamous cell lung carcinoma, 11 differently expressed miRNAs for the comparison between adenocarcinoma and SCLC, and 2 miRNAs for the comparison between squamous cell lung carcinoma and SCLC, each with a raw p-value < 0.05. Hsa-let-7c was the only deregulated miRNA in the comparison between NSCLC and SCLC. The respective miRNAs and raw p-values are summarized in Table 1. The most differentially expressed miRNAs were found for comparisons with adenocarcinoma. These results indicate that there are no major differences of the blood-borne miRNA expression pattern between NSCLC and SCLC.

**Table 1:** **Deregulated miRNAs based on raw p-values < 0.05**

MiRNA	Adenocarcinoma vs squamous cell lung cancer	Adenocarcinoma vs SCLC	Squamous cell lung cancer vs SCLC	NSCLC vs SCLC
hsa-miR-296-5p	**0.009293704**	**0.002492158**	-	-
hsa-miR-4286	**-**	-	0.042230044	-
hsa-miR-125b	**-**	**0.028149193**	-	-
hsa-miR-222	**-**	-	0.04703922	-
hsa-miR-1202	**0.007974179**	-	-	-
hsa-miR-30c	**-**	**0.041578614**	-	-
hsa-miR-30d	**0.023430857**	-	-	-
hsa-miR-484	**-**	**0.030142088**	-	-
hsa-miR-191	**0.037639111**	-	-	-
hsa-miR-378*	**0.019775161**	**0.010497338**	-	-
hsa-miR-494	**0.019335653**	-	-	-
hsa-miR-20a	0.013479972	-	-	-
hsa-miR-93*	**0.047492422**	**-**	-	-
hsa-miR-185	**0.021057518**	**0.02356637**	-	-
hsa-miR-93	0.025342321	-	-	-
hsa-miR-550a*	**0.031935527**	**0.021650736**	-	-
hsa-miR-423-3p	**-**	**0.014698713**	-	-
hsa-let-7c	-	0.036109015	-	0.038753591
hsa-let-7f	0.043924552	-	-	-
hsa-let-7 g	0.015803657	-	-	-
hsa-miR-17	0.017017848	-	-	-
hsa-miR-16	0.04337876	-	-	-
hsa-miR-664	**0.029957884**	-	-	-
hsa-miR-26b	0.044474487	-	-	-
hsa-miR-146a	-	0.033986273	-	-
hsa-miR-107	0.031934222	0.023377571	-	-

miRNAs with raw p-value <0.05. bold font = down-regulated in first group of comparison, normal font = up-regulated in first group of comparison.

**Figure 2:**
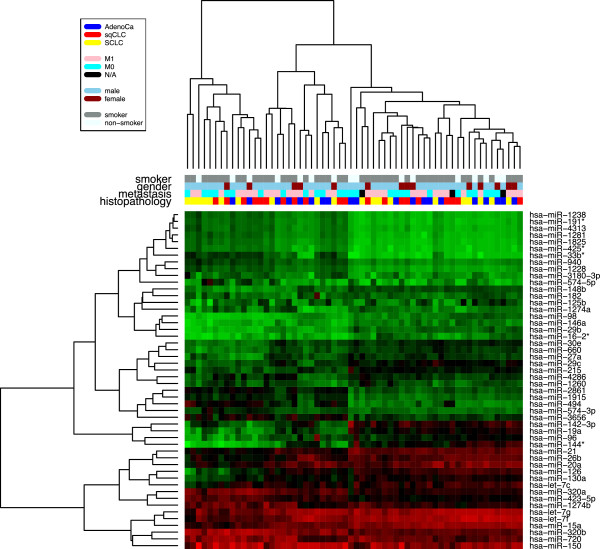
**Heatmap of the 50 miRNAs with highest variance and all samples.** Each column represents a blood sample and each row represents a miRNA. The miRNA expression values are visualized by the red-green color code, where green means low expression and red means high expression. The colored bars under the dendrogram indicate the histological subtype, the smoking status, the gender, and the metastatic state of the respective samples (red = squamous cell lung cancer (sqCLC), blue = adenocarcinoma, yellow = SCLC, pink = metastasis (M1a or M1b), cyan = no metastasis (M0), black = no information on metastasis state, skyblue = male, darkred = female, grey = current or former smoker, white = never-smoker).

The above mentioned findings were largely confirmed when we focused on miRNAs with a fold change of at least 1.5 independent of the p-value. The comparison of adenocarcinoma samples with squamous cell lung cancer samples yielded the highest number of deregulated miRNAs (n = 20) including five miRNAs with a raw p-value <0.05. The comparison between adenocarcinoma and SCLC revealed 17 miRNAs including two miRNAs with p-values <0.05, and the comparison between squamous cell lung carcinoma and SCLC revealed 7 miRNAs including one with a p-value <0.05. The same number of deregulated miRNAs was found for NSCLC vs. SCLC, but none of those 7 miRNAs had a p-value <0.05 (Table 2).Overall, the above data indicate a high degree of similarity between the blood-borne miRNA signatures of the different subtypes of lung cancer. This is also visualized in Figure 2 that shows a heatmap with the 50 miRNAs with highest variance and all samples. There is no clear clustering into the three different histological subtypes.

**Table 2:** **Deregulated miRNAs based on fold changes >1.5**

MiRNA	Adenocarcinoma vs squamous cell lung cancer	Adenocarcinoma vs SCLC	Squamous cell lung cancer vs SCLC	NSCLC vs SCLC
hsa-miR-320b	**1.565908352**	-	1.647174369	-
hsa-miR-320a	**1.682325336**	-	-	-
hsa-miR-16-2*	-	1.556819993	1.584825817	1.547144675
hsa-miR-425*	-	**1.558378991**	-	-
hsa-miR-1825	-	**1.601678188**	-	-
hsa-miR-126	1.891966989	-	-	-
hsa-miR-19a	1.649266976	2.162558226	-	1.578154988
hsa-miR-144*	2.055635088	-	-	-
hsa-miR-1238	-	**1.615812614**	-	-
hsa-miR-1202	***1.794851186***	*-*	-	-
hsa-miR-33b*	-	**3.71072872**	**2.277178734**	**3.455716247**
hsa-miR-3180-3p	-	**1.540492953**	-	-
hsa-miR-21	1.576532119	1.517125311	-	-
hsa-miR-494	***3.238384244***	**2.523949933**	-	**1.604162158**
hsa-miR-125b	-	*1.893914674*	-	1.576963025
hsa-miR-4286	-	-	*1.606159073*	1.505950661
hsa-miR-3651	**1.507544659**	-	-	-
hsa-miR-940	**1.513643787**	-	-	-
hsa-miR-20a	*1.807832821*	1.644649446	-	-
hsa-miR-1228	**1.613581053**	-	-	-
hsa-miR-3162	**1.560608345**	-	-	-
hsa-miR-98	1.657974523	-	-	-
hsa-miR-96	1.65850084	1.519616853	-	-
hsa-miR-191*	-	**1.557935486**	-	-
hsa-let-7b	-	-	1.633347369	-
hsa-let-7a	1.545768531	-	**1.522487952**	-
hsa-let-7 g	*1.580842517*	-	-	-
hsa-miR-142-3p	2.29109294	2.221864576	-	1.563956799
hsa-miR-197	-	**1.506054329**	-	-
hsa-miR-26b	*2.299936662*	-	**1.58391195**	-
hsa-miR-146a	-	*1.681236456*	-	-
hsa-miR-1281	-	**1.501429345**	-	-
hsa-miR-1280	**1.601529185**	-	-	-

miRNAs with fold changes >1.5. bold font = down-regulated in first group of comparison, normal font = up-regulated in first group of comparison, italic font = raw p-value <0.05.

However, we found few miRNAs that were deregulated in the same direction in two or three of the comparisons. The miRNAs hsa-miR-296-5p, hsa-miR-378*, hsa-miR-185, and hsa-miR-550a* were down regulated in adenocarcinoma compared to squamous cell lung carcinoma and in adenocarcinoma compared to SCLC. In the same two comparisons hsa-miR-107 was over-expressed in adenocarcinoma. MiRNA hsa-let-7c was up regulated in adenocarcinoma compared to SCLC and in NSCLC compared to SCLC.

For validation purposes we performed quantitative Real Time PCR (RT-qPCR) on five miRNAs using an independent group of patients, which were not analyzed by the microarray approach. As shown in Additional file 1: Figure S1, we confirmed most of the measured miRNAs.

Next, we analyzed the overlap between the blood-borne miRNAs that were expressed in the different histological subtypes and a set of 24 deregulated miRNAs that was previously shown by our group to separate lung tumor patients and controls with high accuracy [24]. Out of those 24 blood-borne miRNAs, 17 miRNAs were detected in the blood samples of all 20 adenocarcinoma patients, 20 miRNAs in all squamous cell lung carcinoma patients, and likewise 20 miRNAs in all SCLC patients (see Additional file 2: Table S1). The extended overlap of the 24-miRNA signature with each of the three histological subgroups indicates that the signature does not primarily depend on a histological subtype. The same analysis was performed with deregulated miRNAs identified in other studies on lung cancer either on whole blood samples or on tissue specimen. We have to point out that the studies on whole blood were mostly based on the identification of deregulated miRNAs between blood from lung cancer patients and blood from healthy controls or patients with other lung diseases and not on the differentiation of the different histological lung cancer subtypes [22, 26, 28, 29]. Studies that compared miRNAs between histological subgroups of lung cancer did not use whole blood as source of the miRNA but are based on the analysis of FFPE, biopsies, brushing specimens, fine needle aspirations etc. [12–15, 30–32]. Additional file 2: Table S1 gives an overview of this analysis. The majority of miRNAs identified in the above mentioned studies on whole blood samples from lung cancer patients and controls and on tissue samples from different histological lung cancer subgroups were detected in most of the blood samples analyzed in the present study.

### Influence of metastases on the miRNA expression

We investigated whether the dissemination of primary tumor cells into the surrounding and growth of secondary malignancies (metastases) affects the miRNA expression pattern in blood cells. Using miRNAs with raw p-value < 0.05 we were able to differentiate between patients with and without metastases in the adenocarcinoma and the SCLC group with about 75% accuracy and an AUC of 0.73 and 0.82, respectively. The miRNA expression pattern in blood of squamous cell lung cancer patients did no differ due to metastases. Table 3 lists the classification results together with the corresponding miRNAs.

**Table 3:** **Classification results for patients with and without metastases**

	Adenocarcinoma	Squamous cell lung carcinoma	SCLC
accuracy, mean	0.7526	0.5079	0.7526
specificity, mean	0.8	0.4666	0.7792
sensitivity, mean	0.7	0.545	0.7072
AUC, mean	0.73	0.54	0.82
selected miRNAs	hsa-miR-361-5p hsa-miR-3651 hsa-miR-28-5p	hsa-miR-378*	hsa-miR-103
		hsa-miR-423-3p	hsa-miR-328
		hsa-miR-550a*	hsa-miR-15b
			hsa-miR-199a-5p

Classification with linear kernel, 10-fold cross-validation, 20 repetitions using miRNAs with raw p-value < 0.05.

### Effect of cigarette smoke on the miRNA expression

Cigarette smoking is the main risk factor for developing lung cancer. However, a high proportion of adenocarcinoma occurs also in never smokers. We were interested if cigarette smoking has an effect on the overall miRNA expression pattern in patient’s blood. To address this, we compared the miRNA expression profile of smokers and non-smokers in the adenocarcinoma group. After adjustment by the Benjamini-Hochberg approach, no overall significant expression changes were observed due to cigarette smoking among the adenocarcinoma patients. Regarding only miRNAs with raw p-value <0.05, we identified ten miRNAs (hsa-miR-20a, hsa-miR-18b, hsa-miR-20b, hsa-miR-17, hsa-miR-16, hsa-let-7i, hsa-miR-182, hsa-miR-183, hsa-miR-92a, hsa-miR-151-5p) expressed in at least 100% of either the smokers or the non-smokers that were able to separate both groups with 76.8% accuracy and an AUC of 0.93.

## Discussion

An increasing number of studies show that miRNAs from blood can be used as biomarkers for various human diseases. The spectrum of diseases analyzed includes cancer as well as non-cancer diseases. While the majority of these studies analyzed serum or plasma, there are also a considerable number of investigations on miRNA expression in PBMCs or whole blood. Independent of the question whether serum, plasma, PBMCs or whole blood was analyzed, these studies provide strong evidence that blood based miRNA signatures can be used to differentiate between controls and patients. In a comparative analysis of blood-borne miRNA profiles in 14 diseases, we showed recently that miRNA signatures could even differentiate between different diseases of the same organ as for example between lung cancer and COPD with accuracy over 90%. It was also shown that miRNA expression profiles could not only indicate the probability of a diseases but also the probability of the absence of a disease [23].

Tissue based miRNA expression analyses even revealed specific miRNA expression pattern in different histological cancer subtypes, including lung cancer. For example, miRNA expression analysis allowed to correctly differentiating transthoracic needle aspiration biopsy specimens from NSCLC patients in adenocarcinoma and squamous cell lung carcinoma [12]. Sets of two miRNAs accurately discriminated between NSCLC and SCLC and between adenocarcinoma and squamous cell lung carcinoma in bronchial brushing specimens [13]. MiRNA analysis also distinguished NSCLC from SCLC cell lines [15]. A small subset of eight miRNAs classified small pre-operative biopsies into squamous cell carcinoma, non-squamous non–small cell lung cancer, carcinoid, and small cell carcinoma [14]. Interestingly, we found a high overlap of deregulated miRNAs identified in the above mentioned studies on tissue samples from different histological lung cancer subgroups and the miRNAs detected in the blood samples analyzed in the present study. However, there is considerable less evidence that blood-borne miRNA signatures can identify specific subcategories within a disease as for example a specific tumor grading or histological cancer subtypes. As a first attempt to address this question we analyzed three histological subtypes of lung cancer. Lung cancer was chosen since the best possible lung cancer treatment requires accurate subclassification. The clinically most important differentiation is between NSCLC and SCLC as they differ not only histologically but also in their behavior. SCLCs are very fast growing tumors making them the most aggressive form of lung cancer with chemotherapy or radiotherapy or a combination of both as common treatment. NSCLC treatment depends on cancer stage; surgery is the treatment of choice for early stage NSCLC (stage I and II) [3, 4]. However, as about 70% of NSCLCs are diagnosed at advanced stages, systemic therapy (chemo- or targeted therapy) and/or radiation is recommended [5].

Our results show that relatively few blood-borne miRNAs were differentially expressed between patients with adenocarcinoma and with squamous cell lung carcinoma, and even less between NSCLC and SCLC. The similarity between the miRNA signature did not allow a separation between the different histotypes, as it was previously reported for miRNAs that were derived from tumor tissues [12–15, 30–34]. Interestingly, the miRNA hsa-miR-205 that was shown in several publications to distinguish between tissue samples from adenocarcinoma and squamous cell lung cancer patients was not detected in any of the analyzed blood samples [12–15, 31–34]. The results that we obtained for the blood-borne miRNA signatures are, however, consistent with the results recently obtained for miRNA isolated from serum that were also independent from histology [35, 36]. Serum derived miRNA signatures showed a comparable risk of cancer death between patients with adenocarcinoma or squamous cell carcinoma. Likewise, serum derived miRNA signatures showed a comparable risk of cancer death between patients with either stage I, II, or IIIa carcinomas [35]. Lin and colleagues [36] identified two serum miRNAs, namely hsa-miR-126 and hsa-miR-183 that may serve as potential serum biomarkers for metastatic non-small-cell lung cancer. This prompted us to investigate if the miRNA expression pattern of whole blood might also be influenced by metastases. Using a set of three to four miRNAs we were able to differentiate between metastatic and non-metastatic adenocarcinoma and SCLC samples, but this was not possible for squamous cell lung cancer samples. The two miRNAs hsa-miR-126 and hsa-miR-183 identified by Lin et al., were indeed detected in all of our analyzed samples, however, they were not included in our set of miRNAs. A classification with these two miRNAs and our samples was not possible. The three miRNAs that classified between metastatic and non-metastatic adenocarcinoma were expressed in all analyzed blood samples, but were all up-regulated in metastatic adenocarcinoma (between 1.4 and 1.7 fold). These miRNAs have never been related to cancer metastasis in literature. However, hsa-miR-361-5p was identified to be a regulator of VEGFA and thus associated with skin cancer [37]. The four miRNAs that classified between metastatic and non-metastatic SCLC were also expressed in all analyzed blood samples. Two miRNAs were up-regulated and two miRNAs were down-regulated between 1.2 and 1.5 fold in metastatic SCLC. Hsa-miR-328 that was 1.4 fold up-regulated in metastatic SCLC has previously been shown to mediate NSCLC migration and to be associated with NSCLC brain metastasis [38]. In colorectal cancer tissue hsa-miR-103 might promote metastasis by targeting the known metastasis suppressors death-associated protein kinase (DAPK) and Krüppel-like factor 4 (KLF4) [39]. However, in blood of metastatic SCLC patients, this miRNA was slightly down-regulated (1.2 fold). The two other miRNAs hsa-miR-15b and hsa-miR-199a-5p have not been associated with metastasis or lung cancer.

## Conclusions

Our data do not provide evidence that miRNA signatures derived from blood can readily distinguish lung cancer subtypes. Thus, we can conclude that blood-borne miRNA signatures are not suitable replacements for currently used methods for the subtyping of lung cancer. However, as addressed above miRNA signatures can be used to indicate the presence/absence of a disease [23]. In the present study we found a large overlap between the blood-borne miRNAs of each histological group and the previously described blood-borne 24-miRNA signature that separates lung cancer patients form controls [24]. These results indicate that the previously described 24-miRNA signature for lung cancer appears not primarily dependent on histological subtypes. However, some miRNAs might be indicative for the metastatic potential of adenocarcinoma and SCLC. Without symptoms, metastases may only be discovered by imaging techniques like X-ray, CT, or PET that are routinely done every few months. Alternatively, patients might meanwhile benefit from a simple blood test that can indicate the development of a metastasis.

In summary, analyzing miRNA expression in blood cells appears less suitable to define tumor subtypes but to classify human cancer entities and to predict the metastatic potential of certain histological tumor types.

## Materials and methods

### Patients

For the microarray analysis, we obtained 2,5 ml peripheral blood in PAXgene Blood RNA tubes (BD, Franklin Lakes, New Jersey USA) from 60 lung cancer patients, including 40 NSCLC patients (20 adenocarcinoma and 20 squamous cell lung cancer) and 20 SCLC patients from Department of Pneumology, Medical School, Saarland University. For determining the tumor stage the 7th Edition of the UICC TNM classification of malignant tumours was used [40]. The blood samples were drawn before therapy with the exception of 9 patients that were formerly diagnosed with lung cancer and already underwent therapy at that former period. For those patients an overview is given in Table 4. More detailed information on these patients is given in Additional file 3: Table S2.

**Table 4:** **Patient characteristics**

	Adenocarcinoma	Squamous cell lung cancer	SCLC	***Χ*** ^2^p-value
number of samples	20	20	20	
mean age, years (SD)	63.5 (±10.15)	65 (±8.12)	63 (±10.15)	
range				
gender, n				0.911
female	5	5	4	
male	15	15	16	
smoking, n				0.0001989
never-smoker	12	2	2	
current or former smoker	8	18	18	
Staging, n				0.02329
I-II	7	2	1	
III-IV	12	17	18	
unknown	1	1	1	
metastases, n				0.6095
M0	9	10	7	
M1a / M1b	10	9	12	

For the RT-qPCR validation we obtained 2,5 ml peripheral blood in PAXgene Blood RNA tubes (BD, Franklin Lakes, New Jersey USA) from an independent patient cohort (9x adenocarcinoma, 9x squamous cell lung cancer, 5x SCLC) derived from a second institution, i.e., Department of Thoracic Surgery, Voelklingen Heart Center.

Informed consent was obtained from each study subject and the study was approved by the local ethics committee (Ärztekammer des Saarlandes; ID 01/08).

### RNA isolation

Total RNA including miRNA was isolated using the PAXgene Blood miRNA Kit (Qiagen) following the manufacturers recommendations. Isolated RNA was stored at -80C. RNA integrity was analyzed using Bioanalyzer 2100 (Agilent) and concentration and purity was measured using NanoDrop 2000 (Thermo Scientific).

### Microarrays

Microarray analysis was performed according to the manufacturer’s instructions using SurePrint 8x60K Human v16 miRNA microarrays (Agilent, CatNo G4870A) that contain 40 replicates of each of the 1205 miRNAs of miRBase v16 (http://www.mirbase.org/). In brief, a total of 100 ng total RNA was processed using the miRNA Complete Labeling and Hyb Kit to generate fluorescently labeled miRNA. This method involves the ligation of one Cyanine 3-pCp molecule to the 3' end of a RNA molecule with greater than 90% efficiency. First, the RNA is dephosphorylated using Calf Intestinal Alkaline Phosphatase (CIP). After the dephosphorylation step, dimethylsulfoxide (DMSO), which is an effective RNA denaturant, is added to the samples and the RNA is heat denaturated to minimize the effect of structure and sequence differences among miRNAs. Using T4 RNA ligase and a 3´,5´-cytidine bisphosphate which is labelled by a cyanine dye at its 3´ phosphate (pCp-Cy3) miRNA molecules with an additional 3´-cytidine and exactly one cyanine dye on its 3´end are produced. After the labeling reaction, the mixture is dried in a vacuum centrifuge and resuspended in the hybridization mixture containing hybridization buffer and blocking reagent. Then the microarrays were loaded and incubated for 20 h at 55C and 20 rpm. To check if the labeling and hybridization was successful, labeling and hybridization spike-in controls were added in the appropriate steps. After several washing steps microarrays were scanned with the Agilent Microarray Scanner at 3 microns in double path mode. Microarray scan data were further processed using Feature Extraction software to extract signal intensity values from the image file.

### Quantitative Real Time PCR (RT-qPCR)

To validate our microarray data we analyzed the expression of five miRNAs (hsa-let-7 g, hsa-miR-125b, hsa-miR-146a, hsa-miR-20a, hsa-miR-4286) using quantitative Real Time-Polymerase Chain Reaction (RT-qPCR) using a second independent patient cohort, including 9 adenocarcinoma patients, 9 squamous cell lung cancer patients, and 5 SCLC patients. We used the miScript PCR System (Qiagen) for reverse transcription and RT-qPCR. RNA was converted into cDNA using the miScript II Reverse Transcription Kit and the HiSpec Buffer according to the manufacturers´ protocol. The RT-qPCR was performed with the miScript SYBR^®^ Green PCR Kit in a total volume of 20 μl per reaction containing 1 μl diluted cDNA according to the manufacturers´ protocol. RNU48 served as endogenous control.

### Statistical data evaluation

To calculate the total expression value for each miRNA per sample we summed up the gTotalProbeSignals in the feature extraction file, applied quantile normalization to normalize expression values across the arrays using the preprocessCore package of the programming language R, and performed a log_2_ transformation of the data. We carried out parametric *t*-test (unpaired, two-tailed) for each miRNA separately, to detect miRNAs that show different behavior in different groups of blood donors. The resulting p-values were adjusted for multiple testing by Benjamini-Hochberg [41, 42] adjustment. Classification of samples using miRNA patterns was carried out using Support Vector Machines (SVM, [43]) as implemented in the R e1071 package [44] using 20 repetitions of standard 10-fold cross-validation and a subset selection technique based on *t*-test.

## Electronic supplementary material

Additional file 1: Figure S1: Comparison of the results obtained by microarray and by RT-qPCR using two independent patient cohorts. The bars correspond to the fold changes of the tested miRNAs in the respective comparison indicated above the bars (light grey = microarray results, dark grey = RT-qPCR results). (PDF 164 KB)

Additional file 2: Table S1: Analysis of the overlap between the blood-borne miRNAs that were detected in the different histological subtypes in the present study and previously published data on deregulated miRNAs identified in other studies on lung cancer either on whole blood samples or on tissue specimen. (XLSX 64 KB)

Additional file 3: Table S2: Detailed information on the 60 lung cancer patients that were included in the microarray study. (XLSX 135 KB)
