# Supplementary figures and images for: The blood-borne miRNA signature of lung cancer patients is independent of histology but influenced by metastases
Source: Mol Cancer. 2014 Aug 30;13:202. doi: 10.1186/1476-4598-13-202 (PMC4156643; doi:10.1186/1476-4598-13-202)

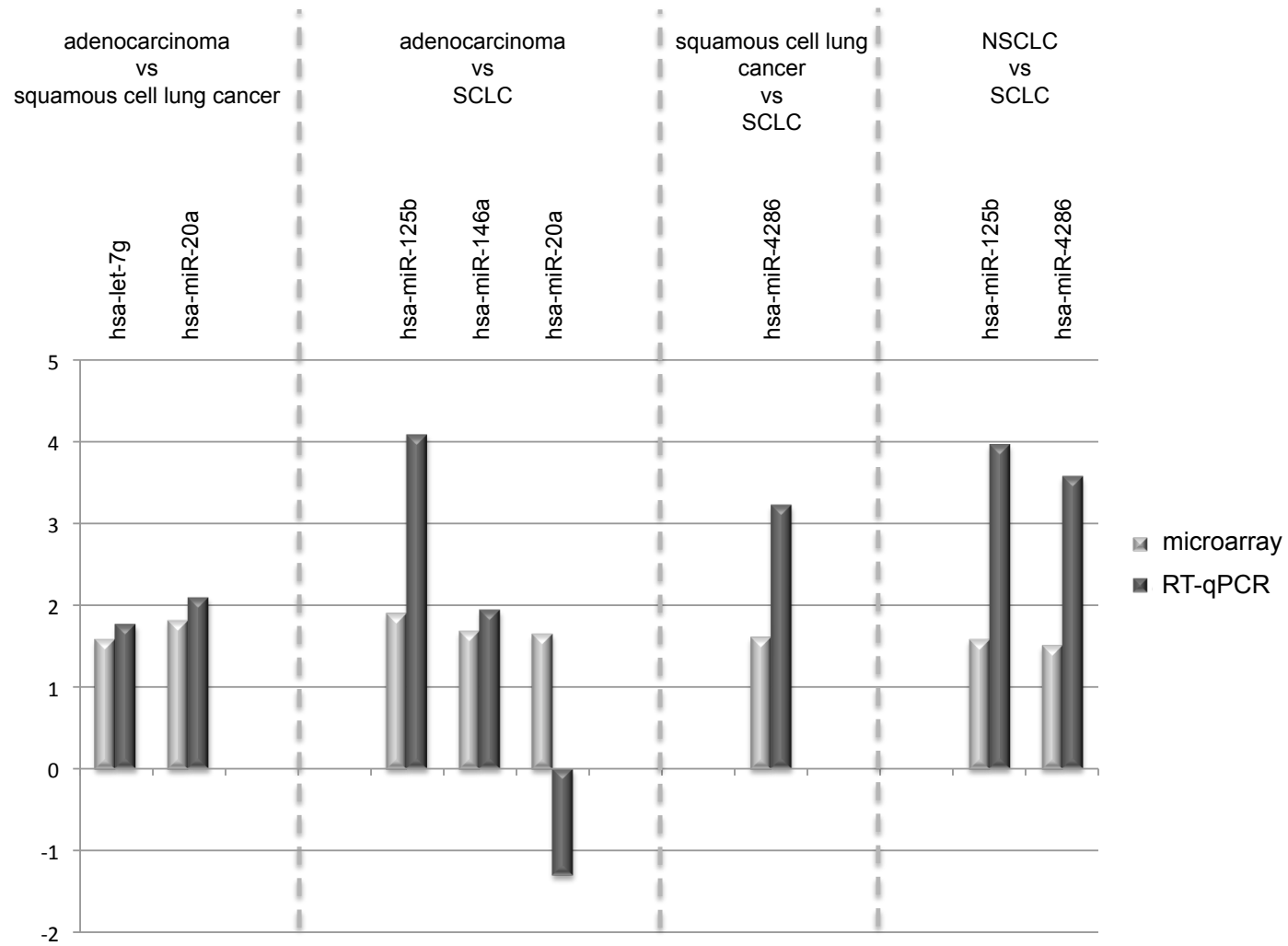

Supplement: Supplementary file 1 — Additional file 1: Figure S1: Comparison of the results obtained by microarray and by RT-qPCR using two independent patient cohorts. The bars correspond to the fold changes of the tested miRNAs in the respective comparison indicated above the bars (light grey = microarray results, dark grey = RT-qPCR results). (PDF 164 KB) [file 12943_2014_1400_MOESM1_ESM.pdf]
